# Superadiabatic Controlled Evolutions and Universal Quantum Computation

**DOI:** 10.1038/srep15775

**Published:** 2015-10-29

**Authors:** Alan C. Santos, Marcelo S. Sarandy

**Affiliations:** 1 Instituto de Física, Universidade Federal Fluminense, Av. General Milton Tavares de Souza s/n, Gragoatá, 24210-346, Niterói, RJ, Brazil; 2 Center for Quantum Information Science & Technology and Ming Hsieh Department of Electrical Engineering, University of Southern California, Los Angeles, California 90089, USA

## Abstract

Adiabatic state engineering is a powerful technique in quantum information and quantum control. However, its performance is limited by the adiabatic theorem of quantum mechanics. In this scenario, shortcuts to adiabaticity, such as provided by the superadiabatic theory, constitute a valuable tool to speed up the adiabatic quantum behavior. Here, we propose a superadiabatic route to implement universal quantum computation. Our method is based on the realization of piecewise controlled superadiabatic evolutions. Remarkably, they can be obtained by simple time-independent counter-diabatic Hamiltonians. In particular, we discuss the implementation of fast rotation gates and arbitrary n-qubit controlled gates, which can be used to design different sets of universal quantum gates. Concerning the energy cost of the superadiabatic implementation, we show that it is dictated by the quantum speed limit, providing an upper bound for the corresponding adiabatic counterparts.

Quantum adiabatic processes are a powerful strategy to implement quantum state engineering, which aims at manipulating a quantum system to attain a target state at a designed time T. In the adiabatic scenario, the quantum system evolves under a sufficiently slowly-varying Hamiltonian, which prevents changes in the populations of the energy eigenlevels. In particular, if the system is prepared in an eigenstate 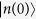 of the Hamiltonian *H* at a time *t* = 0, it will evolve to the corresponding instantaneous eigenstate 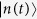 at later times. This transitionless evolution is ensured by the adiabatic theorem, which is one of the oldest and most explored tools in quantum mechanics^1^,^2^,^3^. The huge amount of applications of the adiabatic behavior has motivated renewed interest in the adiabatic theorem, which has implied in its rigorous formulation^4^,^5^,^6^,^7^,^8^,^9^,^10^ as well as in new bounds for adiabaticity^11^,^12^,^13^. In quantum information processing, the adiabatic theorem is the basis for the methodology of adiabatic quantum computation (AQC)^14^, which has been originally proposed as an approach for the solution of hard combinatorial search problems. More generally, AQC has been proved to be universal for quantum computing, being equivalent to the standard circuit model of quantum computation up to polynomial resource-overhead^15^. Moreover, it is a physically appealing approach, with a number of experimental implementations in distinct architectures, e.g., nuclear magnetic resonance^16^,^17^,^18^, ion traps^19^, and superconducting flux quantum bits (qubits) through the D-Wave quantum annealer^20^,^21^,^22^.

Recently, the circuit model has been directly connected with AQC via hybrid approaches^23^,^24^. Then, an adiabatic circuit can be designed based on the adiabatic realization of quantum gates, which allows for the translation of the quantum circuit to the AQC framework with no further resources required. In particular, it is possible to implement universal sets of quantum gates through controlled adiabatic evolutions (CAE)^24^. In turn, CAE are used to perform one-qubit and two-qubit gates, allowing for universality through the set of one-qubit rotations joint with an entangling two-qubit gate^25^,^26^. However, since these processes are ruled by the adiabatic approximation, it turns out that each gate of the adiabatic circuit will be implemented within some fixed probability (for a finite evolution time). Moreover, the time for performing each individual gate will be bounded from below by the adiabatic time condition^4^,^5^,^6^,^7^,^8^,^9^,^10^. For a recent analysis on adiabatic control of quantum gates and its corresponding non-adiabatic errors, see ref. 27.

In order to resolve the limitations of adiabaticity in the hybrid model, we propose here a general shortcut to CAE through simple time-independent counter-diabatic assistant Hamiltonians within the framework of the superadiabatic theory^28^,^29^,^30^,^31^. The physical resources spent by this strategy will be governed by the quantum circuit complexity, but no adiabatic constraint will be required in the individual implementation of the quantum gates. Moreover, the gates will be deterministically implemented with probability one as long as decoherence effects can be avoided. In particular, we discuss the realization of rotation gates and arbitrary n-qubit controlled gates, which can be used to design different sets of universal quantum gates. This analog approach allows for fast implementation of individual gates, whose time consumption is only dictated by the quantum speed limit (QSL) (for closed systems, see refs 32, 33, 34, 35). Indeed, the time demanded for each gate will imply in an energy cost, which increases with the speed of the evolution. In this context, by analyzing the energy-time complementarity, we will show that the QSL provides an energy cost for superadiabatic evolutions that upper bounds the cost of adiabatic implementations.

## Adiabatic quantum circuits

Let us begin by discussing the design of adiabatic quantum circuits as introduced by Hen^24^ through the implementation of quantum gates via CAE.

### Controlled adiabatic evolution

In order to define quantum gates through CAE, we will introduce a discrete bipartite system 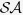 associated with a Hilbert space 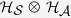. The system 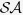 is composed by a target subsystem 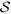 and an auxiliary subsystem 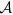, whose individual Hilbert spaces 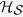 and 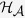 have dimensions 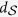 and 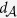, respectively. The dynamics of 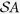 will be governed by a Hamiltonian in the form^24^


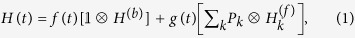


where *f*(0) = *g*(1) = 1, *g*(0) = *f*(1) = 0, and {*P*_*k*_} denotes a complete set of orthogonal projectors over 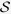, so that they satisfy *P*_*k*_*P*_*m*_ = *δ*_*km*_*P*_*k*_ and ∑_*k*_*P*_*k*_ = 1. Alternatively, we can write Eq. (1) as


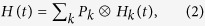


with 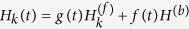 denoting a Hamiltonian that acts on 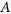. Suppose now that we prepare the system 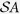 in the initial state 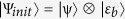, where 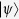 is an arbitrary state of 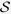 and 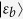 is the (non-degenerate) ground state of *H*^(*b*)^. Then 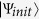 is the ground state of the initial Hamiltonian 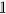
⊗*H*^(*b*)^. By applying the adiabatic theorem^3^,^36^, a sufficiently slowing-varying evolution of *H*(*t*) will drive the system (up to a phase) to the final state


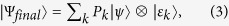


where 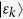 is the ground state of 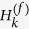
24.

#### Single-qubit unitaries and controlled two-qubit gates

We can perform a single-qubit unitary transformation through a general rotation of an angle *ϕ* around a direction 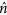 on the Bloch sphere. In this direction, we begin by preparing the system 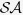, taken here as two qubits, in the initial state 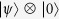, where 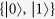 are the computational states of the auxiliary system 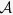. Then, we let the system adiabatically evolve driven by the Hamiltonian^24^


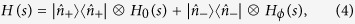


where *H*_0_(*s*) and *H*_*ϕ*_(*s*) are adiabatically-evolved Hamiltonians, whose effect will be restricted to the respective subspaces of the projectors 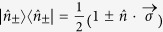, where 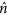 is a unitary vector on the Bloch sphere associated with 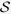 and 
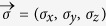
, with {*σ*_*i*_} denoting the set of Pauli matrices. The Hamiltonians are taken as 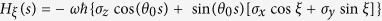, where *ξ* ∈ {0, *ϕ*}, *ωħ* sets the energy scale (*ω* > 0), *θ*_0_ is a constant parameter, and *s* = *t*/*τ* denotes a dimensionless (parametrized) time, with *τ* the total time of evolution. Note then that


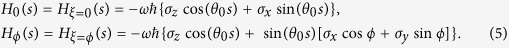


By writing the initial state of 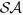 as 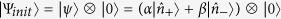, where 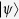 is an arbitrary (not necessarily known) qubit state, the final state 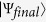 follows from Eq. (3), i.e. 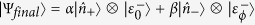. Note that 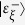 is the ground state of *H*_*ξ*_(*s*), reading 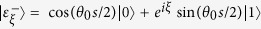, with *ξ* ∈ {0, *ϕ*}. An equivalent form of writing 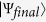 is given by


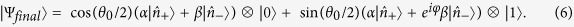


Hence, we have a probabilistic implementation of the rotated target state 
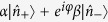
 for an arbitrary angle *ϕ* around an arbitrarily chosen axis 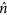, with probability 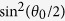. In particular, this probability approximates to one by taking *θ*_0_ ≈ *π*.

In order to perform controlled rotations of a qubit by an angle *ϕ* around a direction 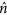, the starting point will be to take the subsystem 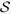 as a two-qubit system and keeping 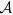 as a single auxiliary qubit. The Hamiltonian is now chosen to be


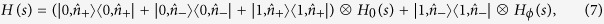


which will govern the evolution of the initial composite state 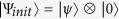, with 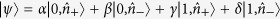. From Eq. (3), the final state of the subsystem 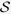 in the limit *θ*_0_ → *π* is now the controlled rotated vector 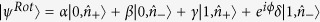. By combining controlled rotations with the single-qubit unitaries discussed above, it is possible to design universal sets of quantum gates through an adiabatic implementation.

## Results

In this Section we present the main results of this work. We start by generalizing the adiabatic implementation of quantum gates proposed in ref. 24 for n-qubit controlled gates. Even though n-qubit controlled gates can be decomposable into one and two-qubit gates (see, e.g. refs 25,37), this implementation implies in an extended class of adiabatic universal gates, e.g. the set {Toffoli, Hadamard}^38^,^39^. Then, we will derive the main result of this work, which is a shortcut for general adiabatic circuits through constant counter-diabatic Hamiltonians, which implies in the possibility of fast analog implementations of quantum circuits. Moreover, we will present an analysis of the quantum speed limit in the context of the energetic cost of the superadiabatic circuit.

### Adiabatic *n*-controlled gates

In order to implement *n*-controlled gates, we define the subsystem 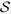 as an (*n* + 1)-qubit system, with the first *n* qubits used as the control register and the last qubit taken as the target register. For the auxiliary system 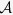, we keep it as a single qubit. Then, we take the initial state as 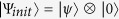, with the subsystem 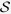 described by


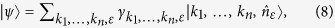


where 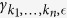 are complex amplitudes, *k*_*l*_ ∈ {0, 1}, 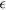 = {±}, and 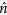 is an arbitrary axis in the Bloch sphere. Here we have written the target qubit in the basis 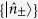, leaving the remaining qubits of 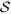 in the computational basis. For simplicity, we will write the states in its decimal representation, i.e.


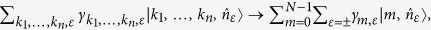


where *N* = 2^*n*^. Then, we let the system evolve driven by the Hamiltonian


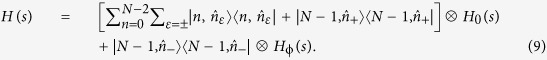


We note that the rotation of the target qubit is expected to be applied if the state of the control system is 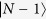. Then, if the Hamiltonian is sufficiently slowly-varying so that we can apply the adiabatic theorem, the system will achieve the final state


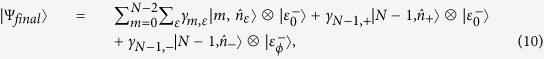


where 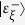 is defined as 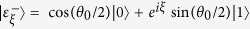 (*ξ* ∈ {0, *ϕ*}). An equivalent form of writing Eq. (10) is


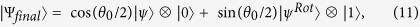


with


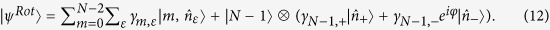


Thus, by performing a measurement over the auxiliary qubit, we find the rotated state 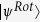 with probability 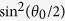. As in the previous case of a rotation controlled by one qubit, this probability can be enhanced to one in the limit *θ*_0_ → *π*. Indeed, this state implies in a rotation of the target qubit in 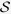 conditioned by the state 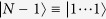 of the control register in 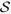. An application of this scheme is the adiabatic implementation of the Toffoli gate, which constitutes an unitary operation implementing an *X* gate over the target qubit if all control qubits are in the state 1, with no effect if any qubit of the control register is in the state 0. This can be easily achieved here by a rotation of an angle *π* around of the direction x, therefore choosing *ϕ* = *π* and 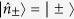, with 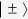 denoting the eigenstates of *σ*_*x*_.

### Shortcut to adiabaticity via counter-diabatic driving

Let us introduce now a shortcut to general CAE through the superadiabatic approach. This will allow for fast piecewise implementation of quantum gates, whose evolution time will not be constrained by the adiabatic theorem. We begin by defining the evolution operator^30^


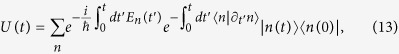


which leads an initial state 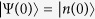 into an evolved state 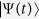 given by


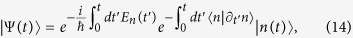


where 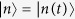 are the eigenvectors of the adiabatic Hamiltonian. Note that this evolution mimics the adiabatic behavior. The Hamiltonian that guides the evolution of the system is the *superadiabatic* Hamiltonian, which reads


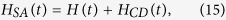


where the additional term *H*_*CD*_(*t*) is the *counter-diabatic* Hamiltonian


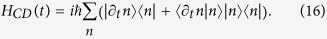


Therefore, a superadiabatic implementation of a dynamical evolution involves the knowledge of the eigenstates of the adiabatic Hamiltonian *H*(*t*), which limits the direct application of the superadiabatic approach in quantum computation. For instance, by adopting the original AQC approach^14^, superadiabatic implementations seem prohibitive, since the whole set of eigenlevels of a many-body Hamiltonian is required. In a similar context, counter-diabatic driving protocols based on realizable settings have been investigated for assisted evolutions in quantum critical phenomena^40^,^41^,^42^. Here, as we shall see, the superadiabatic implementation of universal quantum circuits in the hybrid approach can be promptly achieved, since we deal with the eigenspectrum of piecewise Hamiltonians, which act over a few qubits. It is then appealing to formulate a superadiabatic theory to CAE and then to specify it to the implementation of universal sets of quantum gates. Let us begin by establishing the complete set of eigenstates of the Hamiltonian *H*(*t*) provided by Eq. (2). To this end, consider the eigenvalue equation to each Hamiltonian *H*_*k*_(*t*) given by


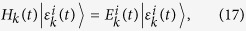


with 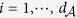. By defining the projectors *P*_*k*_ in Eq. (2) as 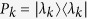, with 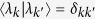 and 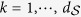, we can write the complete set of eigenstates of *H*(*t*) as


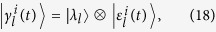


such that 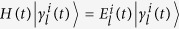. Indeed, from Eq. (2), we have 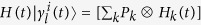

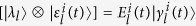
. Note that each projector *P*_*k*_ is associated with a Hamiltonian *H*_*k*_. For instance, for the adiabatic implementation of *n*-controlled gates, we have defined the Hamiltonian *H* in Eq. (9) by linking the set 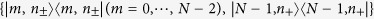 with *H*_0_ and by linking the remaining projector 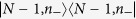 with *H*_*ϕ*_. The next step is to obtain the counter-diabatic Hamiltonian *H*_*CD*_(*t*) that implements the shortcut to the adiabatic evolution of *H*(*t*). In this direction, we use the eigenstates of *H*(*t*) as given by Eq. (18). Then, we get


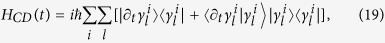


with 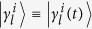. Therefore


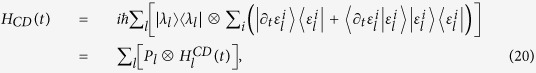


where 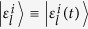 and 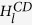 is the counter-diabatic Hamiltonian to be associated with the piecewise adiabatic contribution *H*_*l*_(*t*) acting over subsystem 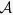, which reads


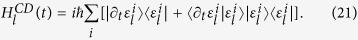


Hence, from Eq. (15), we can implement the shortcut dynamics through the superadiabatic Hamiltonian


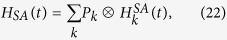


where 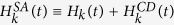 is the piecewise superadiabatic Hamiltonian. Note that the cost of performing superadiabatic evolutions requires the knowledge of the eigenvalues and eigenstates of *H*_*l*_(*t*). For the implementation of general *n*-controlled gates, this is a Hamiltonian acting over a single qubit, which is independent of the circuit complexity. Moreover, we can show that, for an arbitrary *n*-controlled quantum gate, the counter-diabatic Hamiltonians 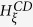 (*ξ* ∈ {0, *ϕ*}) associated with shortcuts to adiabatic evolutions driven by 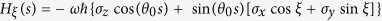, with *s* = *t*/*τ*, are *time-independent* operators given by


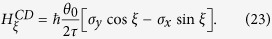


Eq. (23) shows that the implementation of the shortcut can be achieved with a very simple assistant Hamiltonian in the quantum dynamics. Its proof is provided in Section *Methods*.

### Quantum speed limit

It is expected that the shortcut via a counter-diabatic Hamiltonian is faster than the evolution via an adiabatic Hamiltonian, but how much faster can it be? To answer this question, we shall take a lower bound to the time evolution in quantum dynamics as provided by the *quantum speed limit* (QSL). We will consider a closed quantum system evolving between arbitrary pure states 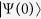 and 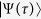. Since the evolution is driven by a time-dependent superadiabatic Hamiltonian *H*_*SA*_(*t*), we will take the generalized Margolus-Levitin bound^33^ derived by Deffner and Lutz^35^, which reads


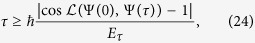


where 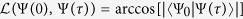 is the Bures metric for pure states^26^ and


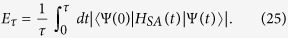


For superadiabatic evolutions, the initial state 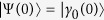 evolves to 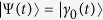, where 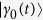 denotes the instantaneous ground state of the adiabatic Hamiltonian *H*(*t*). By using the parametrized time *s* = *t*/*τ*, we can show from Eqs. (24) and (25) that the total time *τ* that mimics the adiabatic evolution within the superadiabatic approach can be reduced to an arbitrary small value. More specifically, the addition of a counter-diabatic Hamiltonian implies into the QSL bound


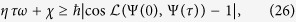


with *η* > 0 and 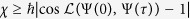, as shown in Section *Methods*. Therefore, the QSL bound reduces to


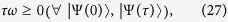


with *τ* and *ω* defined by the superadiabatic Hamiltonian *H*_*SA*_(*t*). This means that the superadiabatic implementation is compatible with an arbitrary reduction of the total time *τ*, which holds *independently* of the boundary states 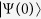 and 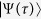. Naturally, a higher energetic cost is expected to be involved for a smaller evolution time *τ*. In particular, saturation of Eq. (27) is achieved for either *τ* → 0 or *ω* → 0, with both cases implying in *τω* → 0. Note that this limit is forbidden in the adiabatic regime for finite *ω*, since the energy gap is proportional to *ħω*, which implies in an adiabatic time of the order *τ*_*ad*_ ∝ 1/*ω*^*n*^, with 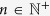
3,4,7,36. Hence, Eq. (27) leads to a flexible running time in a superadiabatic implementation, only limited by the energy-time complementarity.

### The Energetic Cost

Let us show now that time and energy are complementary resources in superadiabatic implementations of quantum evolutions. We shall define the energetic cost associated with a superadiabatic Hamiltonian through


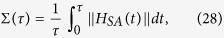


with *H*_*SA*_(*t*) given by Eq. (22) and the norm provided by the Hilbert-Schmidt norm 
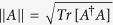
. Since *H*_*SA*_(*t*) is Hermitian, we can write


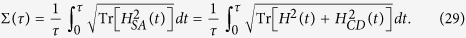


To derive Eq. (29), we have used that Tr({*H*(*t*), *H*_*CD*_(*t*)}) = 0. This can be obtained by computing the trace in the eigenbasis of *H*(*t*) and noticing that the expectation value of *H*_*CD*_(*t*) taken in an eigenstate of *H*(*t*) vanishes, i.e. 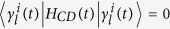. In particular, let us define the energetic cost to the adiabatic Hamiltonian as


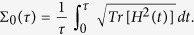


Then, it follows that the energetic cost Σ(*τ*) in superadiabatic evolutions supersedes the energetic cost Σ_0_(*τ*) for a corresponding adiabatic physical process. In order to evaluate Σ(*τ*) we adopt the basis of eigenstates of the adiabatic Hamiltonian *H*(*t*). By using Eq. (18), this yields


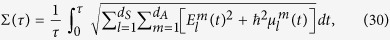


where 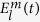 are the energies of the adiabatic Hamiltonian *H*_*l*_(*t*) and


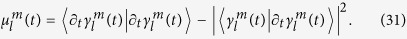


In order to analyze the energetic cost as provided by Eq. (30) for superadiabatic qubit rotation gates, we set 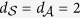 and 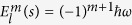 (∀*l*). Moreover, by using Eq. (18), we obtain 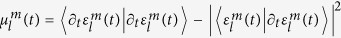, which leads to 
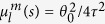
 [See Eqs. (34) and (35) in Section *Methods*]. Hence


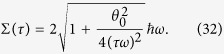


**Figure 1:**
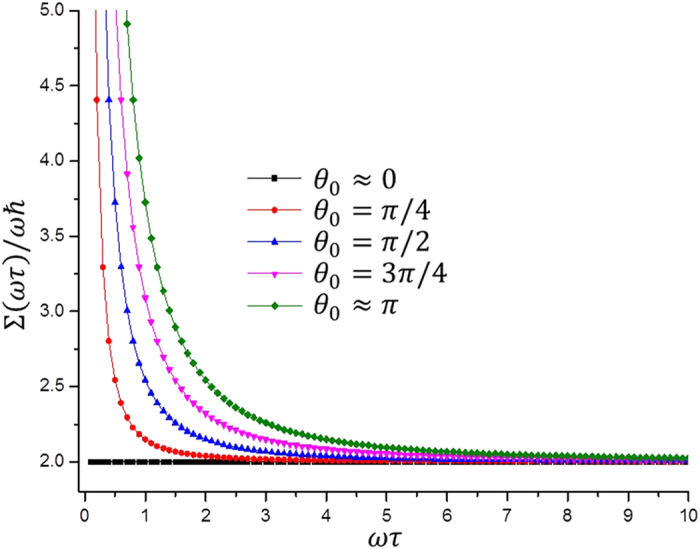
Energetic cost in unities of *ħω* as a function of *ωτ* for different values of *θ*_0_.

We illustrate the behavior of Σ(*τ*) in Fig. 1, where it is apparent that the energetic cost increases inversely proportional to the total time of evolution. In particular, note also that, for a fixed energetic cost, the optimal choice *θ*_0_ → *π* requires a longer evolution. This is because of the fact that, in this case, the final state associated with the auxiliary qubit is orthogonal to its initial state, so it is farther in the Bloch sphere. In the more general case of controlled gates, the analysis is similar as in the case of single-qubit gates. However, we must take into account the number of projectors composing the set {*P*_*k*_}. More specifically, the sum over *l* in Eq. (30) shall run over 1 to 4, which is the number of projectors over the subsystem 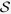. Thus we can show that energetic cost Σ^*CG*^ to implement controlled gates is 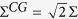.

## Discussion and Conclusion

We have proposed a scheme for implementing universal sets of quantum gates within the superadiabatic approach. In particular, we have shown that this can be achieved by applying a *time-independent* counter-diabatic Hamiltonian in the auxiliary qubit to induce fast controlled evolutions. Remarkably, this Hamiltonian is universal, holding both for performing single-qubit and *n*-controlled qubit gates. Therefore, a shortcut to the adiabatic implementation of quantum gates can be achieved through a rather simple mechanism. In particular, different sets of universal quantum gates can be designed by using essentially the same counter-diabatic Hamiltonian. Moreover, we have shown that the flexibility of the evolution time in a superadiabatic dynamics can be directly traced back from the QSL bound. In this context, the running time is only constrained by the energetic cost of the superadiabatic implementation, within a time-energy complementarity relationship. Implications of the superadiabatic approach under decoherence and a fault-tolerance analysis of superadiabatic circuits are further challenges of immediate interest. In a quantum open-systems scenario, there is a compromise between the time required by adiabaticity and the decoherence time of the quantum device. Therefore, the superadiabatic implementation may provide a direction to obtain an optimal running time for the quantum algorithm while keeping an inherent protection against decoherence. In turn, a basis for such development may be provided by the generalization of the superadiabatic theory for the context of open systems^43^,^44^,^45^,^46^. Concerning error-protection, it may also be fruitful the comparison of our approach with non-adiabatic holonomic quantum computation, where non-adiabatic geometric phases are used to perform universal quantum gates (see, e.g. recent proposals in refs 47,48). Moreover, the behavior of correlations such as entanglement may also be an additional relevant resource for superadiabaticity applied to quantum computation. These investigations as well as experimental proposals for superadiabatic circuits are left for future research.

## Methods

### Time-independent counter-diabatic Hamiltonians for *n*-controlled gates

Let us explicitly design here the superadiabatic implementation of controlled evolutions for piecewise Hamiltonians *H*_*ξ*_(*s*) as provided by Eqs. (5). To this end, consider the eigenvalue equation


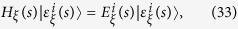


with *ξ* = {0, *ϕ*}, where


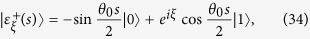



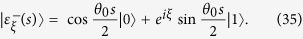


From Eq. (18), it follows that the eigenstates for the adiabatic Hamiltonian *H*(*s*) governing the composite system 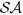 are given by the sets 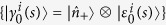 and 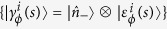 associated with the set of eigenvalues 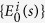 and 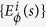, respectively. By evaluating the eigenvalues of *H*_0_(*s*) and *H*_*ϕ*_(*s*), we obtain that their spectra are equal, being provided by 
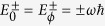
. Thus, *H*(*s*) exhibits doubly degenerate levels, with 
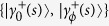
 and 
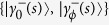
 associated with levels *E*^+^ = *ωħ* and *E*^−^ = −*ωħ*, respectively. By using now Eqs. (34) and (35), we obtain 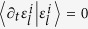, for any *i* = {±} and *ξ* = {0,  *ϕ*}. Then, from Eq. (20), we obtain that the counter-diabatic hamiltonian is 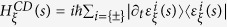, which leads to the time-independent counter-diabatic Hamiltonian given by Eq. (23). The extension to the case of *n*-controlled gates can be achieved as follows. From Eq. (18), the eigenstates of *H*(*s*) read


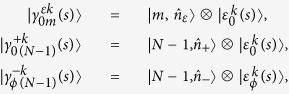


where 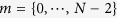, *ε*, *k* = {±} and *ξ* = {0, *ϕ*}. By computing the eigenvalues of *H*(*s*), we obtain that the spectrum of *H*(*s*) is (2*N*)-degenerate, with 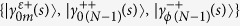 and 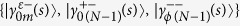 associated with the levels *E*^+^ = *ωħ* and *E*^−^ = −*ωħ*, respectively. By using these results into Eq. (20), we obtain that the counter-diabatic piecewise Hamiltonian 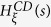 is given by Eq. (23). Hence, the implementation any *n*-controlled gate is achieved through a time-independent counter-diabatic Hamiltonian.

### Quantum speed limit for superadiabatic evolutions

Let us apply here the QSL bound to superadiabatic evolutions. By using the fact than the 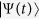 evolves in the ground state 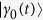 of *H*(*t*) and that *H*_*SA*_(*t*) is given by Eq. (15), we have


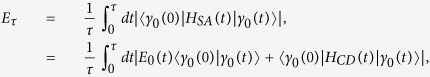


where *E*_0_(*t*) is the instantaneous ground state energy of *H*(*t*). Now we use Eq. (16) and the inequality 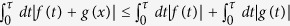, which yields


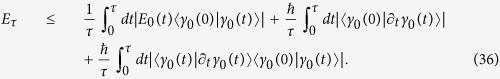


By using the parametrized time *s* = *t*/*τ*, we obtain


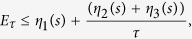


where 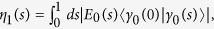 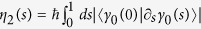 and 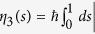 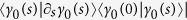. Since the ground state energy for the adiabatic Hamiltonian *H*(*s*) in the case of *n*-controlled gates is *E*_0_(*s*) = −*ωħ* [see Eqs. (5) and (9)], we write *η*_1_(*s*) = *ωη*(*s*), with 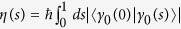. Moreover, we define *χ*(*s*) ≡ *η*_2_(*s*) + *η*_3_(*s*). Then


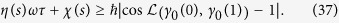


Let us now analise the term *χ*(*s*). First, note that 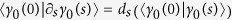. Then, we use that 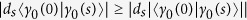 (see proof in ref. 35), which yields


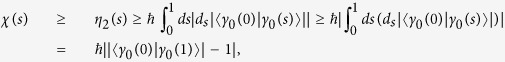


where we have used the inequality 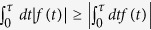. From the definition of the Bures metric, we have 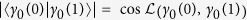. Hence, 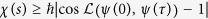, which implies into Eq. (27).

## Additional Information

**How to cite this article**: Santos, A. C. and Sarandy, M. S. Superadiabatic Controlled Evolutions and Universal Quantum Computation. *Sci. Rep.* **5**, 15775; doi: 10.1038/srep15775 (2015).
